# Curcumin improves the therapeutic efficacy of Listeria^at^-Mage-b vaccine in correlation with improved T-cell responses in blood of a triple-negative breast cancer model 4T1

**DOI:** 10.1002/cam4.94

**Published:** 2013-07-02

**Authors:** Manisha Singh, Ilyssa Ramos, Denise Asafu-Adjei, Wilber Quispe-Tintaya, Dinesh Chandra, Arthee Jahangir, Xingxing Zang, Bharat B Aggarwal, Claudia Gravekamp

**Affiliations:** 1 Department of Microbiology and Immunology, Albert Einstein College of Medicine1300 Morris Park Avenue, Bronx, New York, 10461; 2 Cytokine Research LaboratoryDepartment of Experimental Therapeutics, The University of Texas M. D. Anderson Cancer CenterHouston, Texas, 77054

**Keywords:** Cancer vaccines, curcumin, metastases, T cells, triple-negative breast cancer

## Abstract

Success of cancer vaccination is strongly hampered by immune suppression in the tumor microenvironment (TME). Interleukin (IL)-6 is particularly and highly produced by triple-negative breast cancer (TNBC) cells, and has been considered as an important contributor to immune suppression in the TME. Therefore, we hypothesized that IL-6 reduction may improve efficacy of vaccination against TNBC cancer through improved T-cell responses. To prove this hypothesis, we investigated the effect of curcumin, an inhibitor of IL-6 production, on vaccination of a highly attenuated *Listeria monocytogenes* (Listeria^at^), encoding tumor-associated antigens (TAA) Mage-b in a TNBC model 4T1. Two therapeutic vaccination strategies with Listeria^at^-Mage-b and curcumin were tested. The first immunization strategy involved all Listeria^at^-Mage-b vaccinations and curcumin after tumor development. As curcumin has been consumed all over the world, the second immunization strategy involved curcumin before and all therapeutic vaccinations with Listeria^at^-Mage-b after tumor development. Here, we demonstrate that curcumin significantly improves therapeutic efficacy of Listeria^at^-Mage-b with both immunization strategies particularly against metastases in a TNBC model (4T1). The combination therapy was slightly but significantly more effective against the metastases when curcumin was administered before compared to after tumor development. With curcumin before tumor development in the combination therapy, the production of IL-6 was significantly decreased and IL-12 increased by myeloid-derived suppressor cells (MDSC), in correlation with improved CD4 and CD8 T-cell responses in blood. Our study suggests that curcumin improves the efficacy of Listeria^at^-Mage-b vaccine against metastases in TNBC model 4T1 through reversal of tumor-induced immune suppression.

This study is focused on improving cancer vaccination by reducing immune suppression. Here we demonstrate that curcumin improves vaccine efficacy of Listeria-Mage-b by converting myeloid-derived suppressor cells into an immune stimulating phenotype, that is, through reducing IL-6 and increasing IL-12 production, in correlation with improved T cell responses and a dramatic reduction in the number of metastases. The novel results of this study may be a platform for improvement of other cancer vaccines by curcumin.

## Introduction

Triple-negative breast cancer (TNBC), defined as tumors lacking estrogen receptor (ER), progesterone receptor (PR), and HER2/neu accounts for about 20% of all breast cancers, and is particularly increased in black women 1. Women with TNBC represent high-grade tumors that are large and commonly associated with regional node metastases, and recur at distant sites, especially within the first 5 years of diagnosis 2. The absence of any specific targeted therapy for TNBC or basal subtype limits the therapeutic options to cytotoxic therapy 3–4, indicating the need for new therapeutic approaches. Immunotherapy may be our best and most benign option for preventing or curing TNBC. However, immune suppression in the tumor microenvironment (TME) remains as a potential limitation to immunotherapy. Myeloid-derived suppressor cells (MDSC) are one of the most important players in mediating TME-associated immune suppression, with tumor-associated macrophages (TAM), Tregs, and M2 macrophages also playing a role 5–8. Interleukin (IL)-6 is one of such immune suppressive cytokines that is frequently and highly produced by metastatic breast cancers in humans and mice, and particularly by TNBC 9,10. TNBC are enriched for stem-like breast cancer cells (CD44+/CD24−/low), which are typically aggressive and highly resistant to current therapies 12–15. These stem-like breast cancer cells produce high levels of IL-6, and have the capacity to metastasize 16. Moreover, IL-6 is capable of converting dormant breast cancer cells into an actively growing tumor.

IL-6 is a potent regulator of dendritic cell (DC) differentiation in vivo, and is able to turn on the expression of signal transducer and activator of transcription (STAT)3 in DC 17. However, high levels of STAT3 can prevent DC from maturation and subsequent presentation of antigens 18. This in turn may lead to T-cell unresponsiveness. In a previous study, we found high levels of IL-6 produced by breast cancer cells and by immune cells in their TME in an aggressive TNBC mouse model 4T1 19. This IL-6 strongly reduced T-cell responses to Mage-b, but elimination of IL-6 using anti-IL-6 antibodies restored T-cell responses to Mage-b in vitro 20.

Agents that are able to inhibit IL-6 are of great value for immunotherapies against TNBC and other IL-6-producing cancers. One such agent could be curcumin. Curcumin (diferuloylmethane), a polyphenol derived from the plant *Curcumina longa*, commonly called turmeric, has a broad anticancer effect through downregulating transcription factor NFkB thereby affecting downstream genes such as c-myc, Bcl-2, COX-2, NOS, Cyclin D1, TNFα, and MMP9 21. Curcumin is also known for reducing immune suppressive cytokines such as IL-6 through the NFkB pathway 22. It has been shown that curcumin improves therapeutic efficacy of doxorubicin or of B16-R lysate against B16-R melanoma in mice, and that curcumin prevents tumor-induced T cell apoptosis in mice 23–24. In a previous study, we developed a Listeria^at^-based vaccine expressing tumor-associated antigen (TAA) Mage-b 20. Mage-b is homologous to Mage-a 25, and its human homologue MAGE-A is expressed in 26% of the TNBC 26. Vaccination with Listeria^at^-Mage-b showed to be highly effective against metastatic breast cancer in a TNBC model 4T1 in a preventive setting 20. However, Listeria^at^-Mage-b was less effective in a therapeutic setting because of immune suppression in the TME. Here, we demonstrate that curcumin improved therapeutic efficacy of Listeria^at^-Mage-b by reducing the production of IL-6 and increasing the production of IL-12, in correlation with improved T-cell responses in blood of the TNBC 4T1 model. Most important, we found a dramatic effect of the combination therapy on the metastases without having side effects. The results of this study may provide new opportunities to improve efficacy of other types of vaccines and/or against other IL-6-producing cancers.

## Material and Methods

### Mice

Normal female BALB/c (3-month-old) mice were obtained from Charles River and maintained in the animal husbandry facility Albert Einstein College of Medicine according to the Association and Accreditation of Laboratory Animal Care (AACAC) guidelines. All mice were kept under Bsl-2 condition as required for Listeria vaccinations.

### Cells and cell culture

The TNBC 4T1 cell line, derived from a spontaneous mammary carcinoma in a BALB/c mouse 27, was cultured in Dulbecco's modified Eagle's medium (DMEM) supplemented with 10% fetal bovine serum (FBS), 1 mmol/L mixed nonessential amino acids, 2 mmol/L l-glutamine, insulin (0.5 USP units/mL), penicillin (100 units/mL), and streptomycin (100 μg/mL).

### Listeria^at^-based vaccine

In this study, a highly attenuated *Listeria monocytogenes* (Listeria^at^) has been used for the delivery of TAA Mage-b in vivo, as described previously 20. The Listeria^at^ plasmid pGG-34, expresses the positive regulatory factor A (prfA) and one of the virulence genes Listeriolysin O (LLO) 28. The coding region for the C-terminal part of the LLO (cytolytic domain that binds cholesterol in the membranes) protein in the plasmid has been deleted, but Listeria^at^ is still able to escape the vacuole 29. Mutations have been introduced into the prfA gene and in the LLO, which further reduced the pathogenicity of the Listeria^at^ 28. The background strain XFL-7 lacks the prFA gene and retains the plasmid in vitro and in vivo 29. Listeria^at^-Mage-b, expressing nucleotide fragment 311–660 of mouse Mage-b, was developed earlier in our laboratory 20.

### Curcumin

As indicated in the text below, a dose of curcumin (95% curcuminoid) (Alfa Aesar, Ward Hill, MA) of 0.8 or 2 g/kg (20 or 50 mg/mouse) in olive oil was administered orally. Piperine (black pepper) of 20 mg/kg (0.48 mg/mouse) was added to the olive oil in all studies with curcumin. Piperine improves the bioavailability with 2000%, and has been successfully used in humans and animals 30. Piperine is a known inhibitor of hepatic and intestinal glucuronidation, a process that breaks down curcumin in vivo 31–32.

### Immunization and tumor challenge

In this study, two different immunization protocols were tested. The first immunization protocol consisted of three therapeutic immunizations with Listeria^at^-Mage-b and curcumin. Briefly, mice received 0.5 × 10^5^ 4T1 tumor cells in the mammary fat pad on day 0, then 0.5 × 10^7^ CFU of Listeria^at^-Mage-b, or Listeria^at^ or saline intraperitoneally (ip) on days 2, 9, and 16, and finally curcumin orally (50 mg curcumin + 0.48 mg black pepper in olive oil/mouse) on days 4, 5, 6, 11, 12, and 13 (Immunization protocol A). All mice were euthanized on day 17 and analyzed for the number of metastases and tumor growth. All untreated 4T1 mice developed a primary tumor in the mammary fat pad that extended to the chest cavity lining and metastasized predominantly to the mesenteric lymph nodes (MLN), and less frequently to the diaphragm, portal liver, spleen, and kidneys within 14 days (metastases were visible as nodules and counted by eye) as described previously 20_._

The second immunization protocol consisted of three therapeutic immunizations with Listeria^at^-Mage-b, but curcumin was administered before tumor development. Briefly, mice received curcumin orally (50 mg curcumin + 0.48 mg black pepper in olive oil/mouse) on days 0, 1, and 2, then 0.5 × 10^5^ 4T1 tumor cells in the mammary fat pad on day 5, and finally three therapeutic immunizations (ip) with 1 × 10^4^ CFU Listeria^at^-Mage-b, Listeria^at^, or saline on days 8, 11, and 14 (Immunization protocol B). All mice were euthanized on day 16 and analyzed for metastases and tumor growth as described above.

### Flow cytometry analysis

Cells were isolated from spleen and blood as described previously 33. Briefly, red blood or spleen cells were lysed according to standard protocols, and the remaining leukocyte population was used for analysis. Single cell suspensions were also obtained from primary tumors using GentleMacs combined with a mild treatment of the cells using Collagenase, Dispase, and DNAse I, according to the manufacturer's instructions (Miltenyi Biotec Inc, Auburn, CA).

Cells were first incubated with an Fc blocker (anti-CD16), and subsequently with the antibodies for the identification of different cell types. For MDSC, anti-CD11b and -Gr1 antibodies were used. CD11b^+^Gr1^low^ represents monocytic MDSC (mMDSC), and CD11b^+^Gr1^high^ granulocytic MDSC (gMDSC). Anti-CD8 antibodies were used to identify CD8 T cells and anti-CD4 to identify CD4 T cells. Anti-CD45 antibodies were used to identify the leukocyte population in the primary tumors. To detect the production of intracellular lymphokines the cytofix/cytoperm kit from Pharmingen (San Diego, CA) according to the manufacturer's instructions, and antibodies to IL-6, IL-12, and IFNγ were used. Appropriate isotype controls were used for each sample. Depending on the sample size, 10,000–500,000 cells were acquired by scanning using a Fluorescence Activated Cell sorter (flow cytometry) (BD-FACS-Calibur, Franklin Lakes, NJ), and analyzed using Flojo software as described previously 33. Cell debris and dead cells were excluded from the analysis based on scatter signals and use of Fixable Blue or Green Live/Dead Cell Stain Kit (Invitrogen, Grand Island, NY). In blood and spleens, MDSC were analyzed in the total live gated leukocyte population, and T cells in the total live gated lymphocyte population. In the tumor cell suspension, MDSC and T cells were analyzed in the total live gated CD45^+^ (leukocyte) population. All antibodies were purchased from BD Biosciences (San Diego, CA) Pharmingen.

### Cell proliferation, mitotic index, and apoptosis

#### Cell proliferation

4T1 cells (2000 cells in 0.1 mL) were cultured with different doses of curcumin in dimethyl sulfoxide (DMSO) for 72 h, then cell viability was analyzed by 3-(4, 5-dimethylthiazolyl-2)-2, 5-diphenyltetrazolium bromide (MTT) method using a microtiter plate reader at a wave length of 570 nm.

#### Mitotic index

Sections of 1 mm thick of primary tumors of mice treated with Listeria^at^-Mage-b and curcumin or with saline were stained with hematoxylin and eosin (H and E) and subsequently analyzed for the number of cells in mitosis by light microscopy.

#### Apoptosis

Early and late apoptosis was analyzed by Annexin-V and TUNEL assay, respectively. For the Annexin-V assay, 4T1 tumor cells were cultured with or without 100 μmol/L of curcumin for 24 h, and subsequently incubated with Annexin-V antibodies (BD Biosciences), for the detection of apoptosis. For the TUNEL Assay, the ApoTag^®^ In Situ Apoptosis detection (Millipore, Billerica, MA) was used. Briefly, slides were deparaffinized through graded alcohols to PBS. TUNEL staining was performed using ApopTag^®^ In Situ Apoptosis Detection Kit (Millipore). Briefly, samples were Proteinase K digested (20 μg/mL) for 15 min at room temperature. Endogenous peroxidases were blocked using 3% H_2_O_2_ for 5 min at RT. Samples were washed and placed in Equilibration Buffer for 10 sec followed by TdT enzyme incubation in reaction buffer for 1 h at 37°C. Samples were incubated in the Anti-Digoxigenin, washed and developed using DAB (3, 3′-diaminobenzidine). Slides were briefly counterstained in hematoxylin and mounted using Permount (Fisher Scientific, Pittsburgh, PA). From each tissue, two sections were analyzed, and from each section the number of apoptotic cells in 10 fields were counted by light microscopy. The TUNEL assay and Mitotic Index analyses were performed in the Laboratory of Dr. Rani Sellers, Director of Histology and Comparative Pathology Core Facility, Albert Einstein College of Medicine.

### Pathological examination

All pathological analyses were performed by Dr. Rani Sellers, Director of Histology and Comparative Pathology Core Facility, Albert Einstein College of Medicine. Briefly, normal tissues such as kidneys, heart, lungs, liver, and spleen were fixed in 10% formaldehyde for 48 h and then kept in 70% ethanol until use. Sections of 1 mm thick were stained with H and E, and analyzed for pathological damage by light microscopy.

## Results

### Curcumin administered after tumor development significantly improved therapeutic effect of Listeria^at^-Mage-b in the 4T1 model

**Figure 1:**
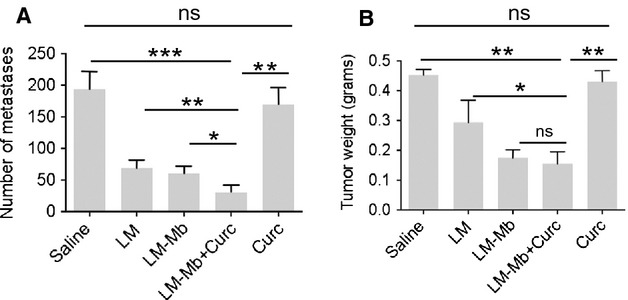
Significant reduction in the number of metastases by therapeutic immunizations with Listeria^at^-Mage-b and curcumin in 4T1 tumor-bearing mice. BALB/c mice were immunized with Listeria^at^-Mage-b and treated with curcumin after tumor development (Immunization protocol A), and analyzed for the frequency of metastases (A) and tumor weight (B). This experiment was performed two times with five mice per group. Average of two experiments. Mann–Whitney *P *<* *0.05 is significant. *P <* *0.05, ** *P* < 0.01, *** *P* < 0.001, ****P < 0.0001; ns, not significant. All groups were compared to LM-Mb+Curc. In addition, curcumin alone was compared to the saline group.

Here, we tested whether curcumin could improve the efficacy of Listeria^at^-Mage-b vaccination in the model 4T1. Listeria^at^-Mage-b and curcumin were alternately administered after tumor development (Immunization protocol A). As shown in Figure 1A, the number of metastases in the mice that received Listeria^at^-Mage-b and curcumin was significantly lower compared to all control groups. Also the tumor weight in the mice that received Listeria^at^-Mage-b and curcumin was significantly lower than in the mice that received Listeria^at^ or curcumin alone, but not compared to the mice that received Listeria^at^-Mage-b alone (Fig. 1B). Curcumin alone had no significant effect on the tumor weight or metastases compared to the saline group.

### Curcumin administered before tumor development also significantly improved therapeutic effect of Listeria^at^-Mage-b in the 4T1 model

**Figure 2:**
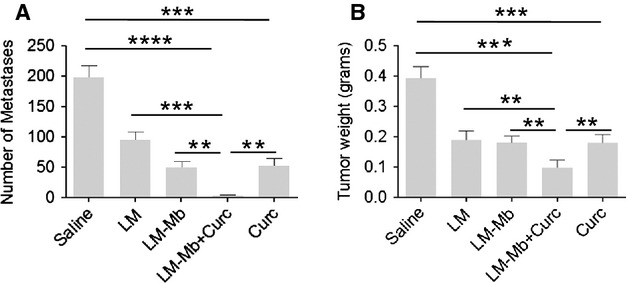
Significant reduction in the number of metastases by preventive administration of curcumin followed by therapeutic immunization with Listeria^at^-Mage-b in 4T1 tumor-bearing mice. BALB/c mice were treated with curcumin before tumor development and immunized with Listeria^at^-Mage-b after tumor development (Immunization protocol B), and analyzed for the frequency of metastases (A) and tumor weight (B). This experiment was performed three times with five mice per group. Average of three experiments. Mann–Whitney *P *<* *0.05 is significant. *P <* *0.05, **P < 0.01, ***P < 0.001, ****P < 0.0001; ns, not significant. All groups were compared to LM-Mb+Curc. In addition, curcumin alone was compared to the saline group.

As curcumin is frequently used in food all over the world, we tested whether curcumin could improve therapeutic vaccine efficacy of Listeria^at^-Mage-b when consumed before tumor development (Immunization protocol B). Here, we used a low dose of Listeria^at^-Mage-b (10^4^ CFU) at a high frequency (every 3 days; four times totally) in order to obtain a continuous delivery of Listeria^at^-Mage-b in vivo without having side effects. Using this immunization protocol, the number of metastases in the mice that received Listeria^at^-Mage-b and curcumin was significantly decreased compared to all control groups (Fig. 2A). Also the tumor weight in the mice that received Listeria^at^-Mage-b and curcumin was significantly lower compared to all control groups (Fig. 2B). Curcumin alone had also a significant effect on the metastases and primary tumors compared to the saline group (Fig. 2B). The growth kinetics of the primary tumors was analyzed as well in mice that received Listeria^at^-Mage-b and curcumin, and confirmed the results shown in Figure 2B; that is, on day 14 the tumor size in mice that received Listeria^at^-Mage-b and curcumin was significantly lower compared to all other control groups (Fig. S1).

**Figure 3:**
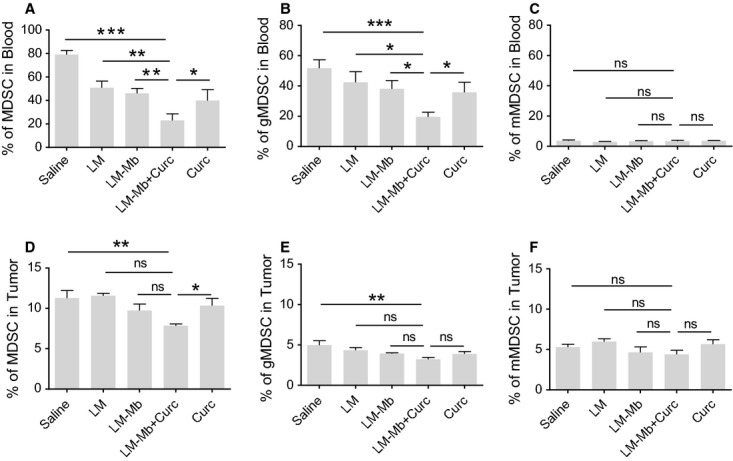
The effect of Listeria^at^-Mage-b and curcumin on MDSC in 4T1 tumor-bearing mice. BALB/c mice were treated with curcumin before tumor development and immunized with Listeria^at^-Mage-b after tumor development (Immunization protocol B), and analyzed for MDSC (CD11b^+^Gr1^+^) (A), gMDSC (CD11b^+^Gr1^high^) (B), and mMDSC (CD11b^+^Gr1^low^) (C) in blood and for MDSC (D), gMDSC (E), and mMDSC (F) in primary tumors using flow cytometry. All groups were compared to Lm-Mb+Curc. Flow cytometry profiles of MDSC of each group (saline, Listeria^at^, Listeria^at^-Mage-b, Listeria^at^-Mage-b and curcumin, curcumin) are presented in Figure S3. This experiment was performed three times with five mice per group. Average of three experiments. Mann–Whitney *P *<* *0.05 is significant. *P <* *0.05, **P < 0.01, ***P < 0.001, **** *P* < 0.0001; ns, not significant.

The combination therapy with curcumin before and Listeria^at^-Mage-b after tumor development was slightly but significantly more effective against the metastases than curcumin and Listeria^at^-Mage-b both after tumor development (Fig. 3A and B); that is, the number of metastases in the combination therapy with curcumin before tumor development was 4 ± 1, and after tumor development 31 ± 12 (Mann–Whitney *P* = 0.0017).

### The effects of Listeria^at^-Mage-b and curcumin on MDSC in vivo

As MDSC strongly contributes to immune suppression in the TME, we analyzed the effect of the combination therapy on MDSC in blood and primary tumors of mice immunized according to immunization protocol B. In total blood, the percentage of MDSC was extremely high (~80%) (Fig. 3A). This percentage was strongly reduced to ~20% by the combination of Listeria^at^-Mage-b and curcumin compared to the saline group, but was also significantly lower compared to all other control groups (Fig. 3A). More detailed analysis showed that gMDSC was predominantly responsible for the strong decrease in percentage of MDSC (Fig. 3B and C). In the primary tumors, the percentage of MDSC was much lower than in blood (~12%), and the effect of Listeria^at^-Mage-b and curcumin on MDSC was much less robust than in blood. The combination therapy slightly but significantly reduced the percentage of MDSC and gMDSC (but not of mMDSC) compared to the saline or curcumin groups only (Fig. 3D–F).

### Curcumin reduced the production of IL-6 in primary tumors and in MDSC

**Figure 4:**
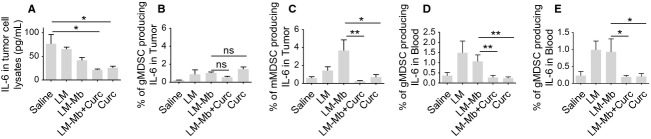
Effects of Listeria^at^-Mage-b and curcumin on IL-6 in 4T1 tumor-bearing mice. Curcumin treatment before tumor development followed by immunizations with Listeria^at^-Mage-b after tumor development (Immunization protocol B), significantly reduced IL-6 levels in primary tumors as shown here by ELISA (A), and the intracellular production of IL-6 by mMDSC in primary tumors (B, C) and by gMDSC and mMDSC in blood (D, E) as shown here by flow cytometry. In A, the curcumin-containing groups were compared to the saline group, while in B, C and E, D, the curcumin-containing groups were compared to Lm-Mb. These experiments were repeated three times with five mice per group, and the results were averaged. Mann–Whitney *P *<* *0.05 is significant *P <* *0.05, **P <* *0.01, ***P <* *0.001, ****P <* *0.0001; ns, not significant.

Here, we analyzed the effect of curcumin on the production of IL-6 in total tumor cell lysates, in MDSC of primary tumors and blood, and in serum of the 4T1 model. In the tumor cell lysates, we found that the curcumin significantly reduced IL-6 levels compared to the saline group (Fig. 4A). In the primary tumor, the IL-6 production in mMDSC was significantly reduced by curcumin compared to Listeria^at^-Mage-b (Fig. 4C), but IL-6 was not produced by gMDSC (Fig. 4B). In blood, the IL-6 production in gMDSC (Fig. 4D) and mMDSC (Fig. 4E) was significantly reduced by curcumin compared to the Listeria^at^-Mage-b group. In serum, IL-6 was undetectable and therefore not shown.

Also Listeria^at^-Mage-b reduced IL-6 levels in the primary tumors (tumor cell lysates) (Fig. 4A), but not in MDSC in blood and primary tumors (Fig. 4B–E). Moreover, Listeria^at^-Mage-b significantly increased the production of IL-6 in sub populations of the MDSC (with an exception of gMDSC in tumors), probably to protect themselves from immune clearance, but as mentioned above curcumin strongly reduced the IL-6 production in both types of MDSC in blood and primary tumor (Fig. 4C–E).

### Curcumin administered before and Listeria^at^-Mage-b after tumor development improved the IL-12 production by MDSC and T-cell responses to Mage-b

**Figure 5:**
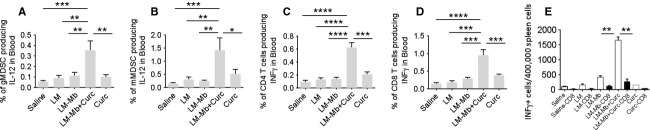
The combination of Listeria^at^-Mage-b and curcumin increased IL-12 production by MDSC and improved T-cell responses in 4T1 tumor-bearing mice. Curcumin treatment before tumor development followed by immunizations with Listeria^at^-Mage-b after tumor development (Immunization protocol B) significantly increased the percentage of gMDSC (A) and mMDSC (B) producing intracellular IL-12 in blood of 4T1 tumor-bearing mice. This correlated with a significant increase in the percentage of CD4 (C) and CD8 T (D) cells producing intracellular IFNγ (activation marker for T cells) in blood of 4T1-tumor-bearing mice as shown here by flow cytometry. CD8 T-cell responses (extracellular production of IFNγ) were also analyzed in the spleen in vitro upon restimulation with Mage-b by ELISPOT, and a significant higher number of CD8 T cells was found in the spleen that received Listeria^at^-Mage-b and curcumin compared to all other groups (E). These experiments were repeated three times with five mice per group, and the results were averaged. Mann–Whitney *P *<* *0.05 is significant. *P <* *0.05, **P <* *0.01, ***P <* *0.001, ****P <* *0.0001.

Here, we analyzed the IL-12 production in subpopulations of gMDSC and mMDSC in blood of mice that received the combination of curcumin before and Listeria^at^-Mage-b after tumor development. A significant increase was found in the percentage of IL-12-producing gMDSC and mMDSC in the combination group compared to all other groups (Fig. 5A and B), but not in the primary tumor (data not shown). These results raised the question whether the lower number of MDSC (Fig. 3), the decreased IL-6 levels (Fig. 4) and increased IL-12 production (Fig. 5A and B) induced by Listeria^at^-Mage-b and curcumin, could improve T-cell responses in vivo. For this purpose, we analyzed the production of IFNγ by CD4 and CD8 T cells in blood and primary tumors in vaccinated and control mice by flow cytometry. IFNγ is a marker for T-cell activation. The cells were analyzed in all groups without restimulation in order to determine whether the T cells were activated in vivo by the combination therapy compared to the control groups. It appeared that the combination of Listeria^at^-Mage-b and curcumin significantly improved the percentage of CD4 and CD8 T cells producing intracellular IFNγ compared to all control groups in blood (Fig. 5C and D), but not in tumors (data not shown). We also analyzed T-cells responses in the spleen upon restimulation with Mage-b in vitro. As shown in Figure 5E, Listeria^at^-Mage-b and curcumin strongly improved the number of CD8 T cells to Mage-b, secreting extracellular IFNγ.

### Curcumin inhibited proliferation of tumor cells and killed tumor cells through apoptosis

**Figure 6:**
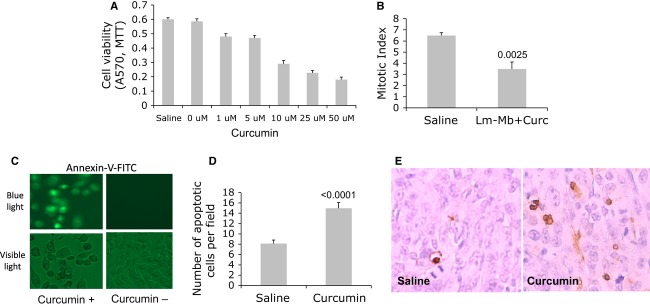
Curcumin inhibited proliferation and killed 4T1 tumor cells through apoptosis. 4T1 tumor cells were cultured with different doses of curcumin for 72 h, and cell viability was analyzed by MTT (A). We also analyzed the Mitotic Index in tumors of mice that received curcumin or saline (B). 4T1 tumor cells were cultured with 100 μmol/L of curcumin in vitro for 24 h, and subsequently incubated with anti-Annexin-V antibodies for the detection of early apoptosis (C). Primary tumors of mice that received curcumin or saline (according Immunization protocol B) were analyzed for the detection of late apoptosis in vivo by the TUNEL assay (D). Apoptotic cells in the primary tumor by the TUNEL assay and light microscopy are shown in (E). Representative of two experiments in A, C, D. Average of two experiments in B and D. *n* = 5 mice per group. Unpaired *t* test *P *<* *0.05 is significant. Magnification light microscopy in C and E is 400×. In A, curcumin was dissolved in DMSO and then diluted to the final concentrations of 1–50 μmol/L. The 0 μmol/L represents DMSO without curcumin.

Several reports describe that curcumin inhibits proliferation and kills tumor cells through apoptosis, including breast tumor cells 34,35. We found that curcumin inhibited the growth of 4T1 tumor cells in vitro (Fig. 6A), and mitosis of the tumor cells in vivo (Fig. 6B). In addition, we found that curcumin killed tumor cells through apoptosis in vitro as shown by Annexin-V (early apoptosis) (Fig. 6C), and in the primary tumors in vivo as shown by the TUNEL assay (late apoptosis) (Fig. 6D). A representative example of apoptotic cells by the TUNEL assay is shown in Figure 6E.

### Listeria^at^-Mage-b is nonpathogenic and curcumin is nontoxic

In a previous study we have shown that Listeria^at^-Mage-b is nonpathogenic 37, while curcumin, consumed through food all over the world, is nontoxic 32. However, the combination of Listeria^at^-Mage-b and curcumin has never been tested. Here, we demonstrate by pathological examination of various normal tissues (as kidney, heart, lungs, liver, and spleen) in tumor-bearing mice that the combination of Listeria^at^-Mage-b and curcumin is nonpathogenic and nontoxic, but primarily activated the innate immune system. Most obvious was the increased extramedullary myeloid hematopoiesis in the spleen and liver of mice that received Listeria^at^-Mage-b and curcumin compared to the saline group. An example of extramedullary myeloid hematopoiesis in the liver is shown in Figure S2. An overview of pathological analysis of normal tissues of tumor-bearing mice that received Listeria^at^-Mage-b and curcumin is shown in Table S1.

## Discussion

Patients with TNBC have the poorest prognosis. One of the main problems of current therapies against TNBC is their inability to target metastases and their high toxicity. They do not respond to therapies that target ER, PR, and HER2/neu because their tumors lack the expression of these receptors/molecules, and other types of therapies such as tyrosine kinase inhibitor sunitinib, targeting vascular endothelial growth factor (VEGF), or therapies targeting c-kit or Flt2, or bevacizumab, a human antibody to VGEF 38–42, are under investigation but with moderate success. In the study presented here, we developed two nontoxic vaccination strategies in a preclinical TNBC mouse model 4T1. We demonstrated that three therapeutic vaccinations with a highly attenuated nonpathogenic Listeria^at^-based vaccine, expressing TAA Mage-b, and nontoxic curcumin significantly reduced the number of metastases compared to Listeria^at^-Mage-b or curcumin alone. Curcumin alone had no significant effect on the primary tumors or metastases. Others described that curcumin killed tumor cells in vitro 43–47. However, tumor cells may react differently to curcumin in vitro than in vivo because in vitro bioavailability and the immune system do not play a role and higher concentrations can be obtained in vitro compared to the in vivo situation. Also, the time point of administering curcumin, the concentration of curcumin, and the type of cancer may determine the antitumor effect of curcumin. For instance, others reported that in the Lewis Lung model, curcumin was not effective against metastases and that the time point of administration of curcumin was important 48.

We also tested three administrations of curcumin before tumor development followed by three immunizations with Listeria^at^-Mage-b after tumor development. This immunization protocol was slightly but significantly more effective against the metastases compared to Listeria^at^-Mage-b and curcumin both after tumor development. Most interestingly, curcumin alone significantly reduced the number of metastases and tumor growth, in contrast to administering curcumin after tumor development. These results suggest that consuming curcumin before cancer develops may provide an advantage over consuming curcumin after cancer develops in the battle against metastatic breast cancer.

Curcumin is known for reducing the production of IL-6 49–50. Here, we demonstrate that curcumin significantly reduced the production of IL-6 in vivo in the primary tumors (tumor cell lysates), and in MDSC of blood and primary tumors. Also Listeria^at^-Mage-b reduced the production of IL-6 significantly in the tumor primary tumors, but IL-6 production was even more reduced by the combination of Listeria^at^-Mage-b and curcumin.

MDSC are important regulators of the immune system in the TME 5–6, and therefore became one of our most important targets in this study. As mentioned above, curcumin reduced the production of IL-6 significantly in MDSC in blood and primary tumors. To our surprise, the combination of Listeria^at^-Mage-b and curcumin significantly increased the production of IL-12 in gMDSC and mMDSC in blood (but not in tumors). It has been reported that IL-12 activates naïve and mature CD4 and CD8 T cells 51–52, which may have happened in this study as well. An interesting observation was that the combination of Listeria^at^-Mage-b and curcumin significantly reduced the number of MDSC (predominantly gMDSC) in blood of the TNBC model 4T1. Various factors may have played a role here. It is possible that MDCS infected with Listeria^at^-Mage-b became a target for Listeria^at^- and Mage-b-specific T-cell and perhaps NK-cell responses because the combination therapy improved these immune responses to Listeria^at^ and Mage-b by reducing IL-6, and increasing IL-12 production. As Listeria^at^ 37 and curcumin kill 4T1 tumor cells directly (this study), it is also possible that the combination therapy prevented the tumor cells from growing in the early phase of treatment, and consequently prevented migration of the MDSC to the TME. We found that curcumin alone decreased the percentage of MDSC in blood (although this effect was much stronger when Listeria^at^-Mage-b was combined with curcumin). Reduction in the percentage of MDSC by curcumin was also found by others in a xenograft model of colon cancer 53. They concluded that reduction in IL-6 production by curcumin reduced the mobilization of MDSC to the primary tumors. Others found that activated T cells might express Fas ligand and induce apoptosis of Fas^+^ MDSC 54. In conclusion, various pathways may lead to the reduction in MDSC and more analysis is required.

The decrease in IL-6 and increase in IL-12 production, the improved CD4 and CD8 T-cell responses in blood and spleen, and the dramatic reduction in the number of metastases by the combination therapy strongly suggest that T-cell responses contributed to the effect on the metastases. However, this strong reduction by the combination therapy is not only an effect of Mage-b-specific T-cell responses. As shown previously, Listeria^at^ exhibits several pathways to kill tumor cells; that is, Listeria^at^ infects tumor cells in vivo and in vitro, and kills tumor cells directly through high levels of reactive oxygen species (ROS) 37. Moreover, we have shown that Listeria^at^-activated CD8 T cells eliminated Listeria^at^-infected tumor cells in vivo 37. In addition, we have shown that curcumin kills 4T1 tumor cells through apoptosis (this study). Therefore, it is most likely that the synergistic effects of the multiple pathways of Listeria^at^-Mage-b and curcumin as described above, are responsible for the overall strong therapeutic effect on the metastases in this TNBC model 4T1.

The therapeutic effect of the combination therapy was strong but less pronounced on the primary tumors compared to the metastases. It is possible that the production of IL-6 was not sufficiently reduced in the primary tumors (IL-6 was reduced in the tumor cell lysates by ~65% by Listeria^at^-Mage-b and curcumin treatment), and other inhibitory cytokines likes TGFβ, which is highly produced by 4T1 tumor cells 55, may play here a role as well. However, most primary tumors can be removed by surgery, radiation, or chemotherapy, while metastases are unresectable and usually chemoresistant despite aggressive and toxic follow-up 56.

The highly attenuated Listeria^at^ of this study is nonpathogenic, and are naturally cleared by the immune system within 3–5 days 37, which is different from wild type Listeria^at^ that multiplies in hepatocytes in the liver or epithelial cells of the gastrointestinal tract 57–58. Moreover, the side effects of the combination therapy of Listeria^at^-Mage-b and curcumin in the 4T1 model were minimal; that is, primarily induction of inflammatory responses in the liver and spleen and no significant findings were observed in other normal tissues such as heart, lungs, and kidneys. Therefore, Listeria^at^-Mage-b and curcumin may be of value as a nontoxic adjuvant therapy, to prevent the development of metastases in TNBC patients that produce IL-6 and express MAGE. This study may be a platform for improvement of other cancer vaccines by curcumin and against other IL-6-producing cancers.

## ELISPOT

Restimulation of spleen cells of vaccinated or control mice was performed as described previously [20]. Briefly, 2×10^5^ spleen cells were transfected with pcDNA3.1-Mage-b plasmid DNA and pCMV-GM-CSF plasmid DNA) (1 μg of each plasmid DNA), using the Nucleofector kit of AMAXA (Gaithersburg, MD). Two days later, the frequency of IFNγ-producing cells was determined by ELISPOT for both restimulation assays according to standard protocols (Pharmingen, San Diego, CA), using an ELISPOT reader (CTL Immunospot S4 analyzer, Cellular Technology Ltd, Cleveland, OH). Spleen cells were depleted for CD8 T cells using magnetic bead depletion techniques according to the manufacturer's instructions (Miltenyi Biotec Inc, Auburn, CA). FACS analysis demonstrated that ≥90% of all CD8 T cells were depleted.

## Supporting Information

**Figure S3.** The effect of Listeriaat-Mage-b and curcumin on MDSC in 4T1 tumor-bearing mice (Flow cytometry profile).
