# Supplementary material for: Curcumin improves the therapeutic efficacy of Listeriaat-Mage-b vaccine in correlation with improved T-cell responses in blood of a triple-negative breast cancer model 4T1
Source: Cancer Med. 2013 Jul 2;2(4):571–82. doi: 10.1002/cam4.94 (PMC3799292; doi:10.1002/cam4.94)

Figure S1

Mann-Whitney

Sal vs LM p=0.2222
Sal vs LM-Mb p=0.0317
Sal vs LM-Mb+Curc p=0.0079
Sal vs Curc p=0.0496

LM-Mb+Curc vs LM-Mb p=0.0162
LM-Mb+Curc vs Curc p=0.0272
$p<0.05$ is significant

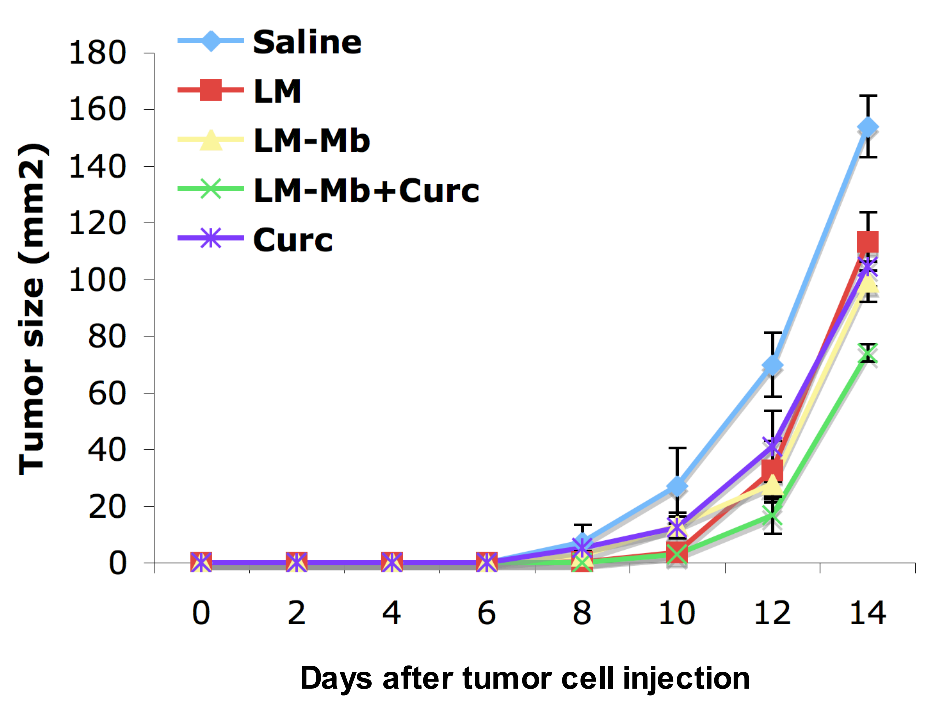

Supplement: Supplementary file 1 [file cam40002-0571-SD1.pdf]
