# Supplementary figures and images for: Curcumin improves the therapeutic efficacy of Listeriaat-Mage-b vaccine in correlation with improved T-cell responses in blood of a triple-negative breast cancer model 4T1
Source: Cancer Med. 2013 Jul 2;2(4):571–82. doi: 10.1002/cam4.94 (PMC3799292; doi:10.1002/cam4.94)

Figure S2

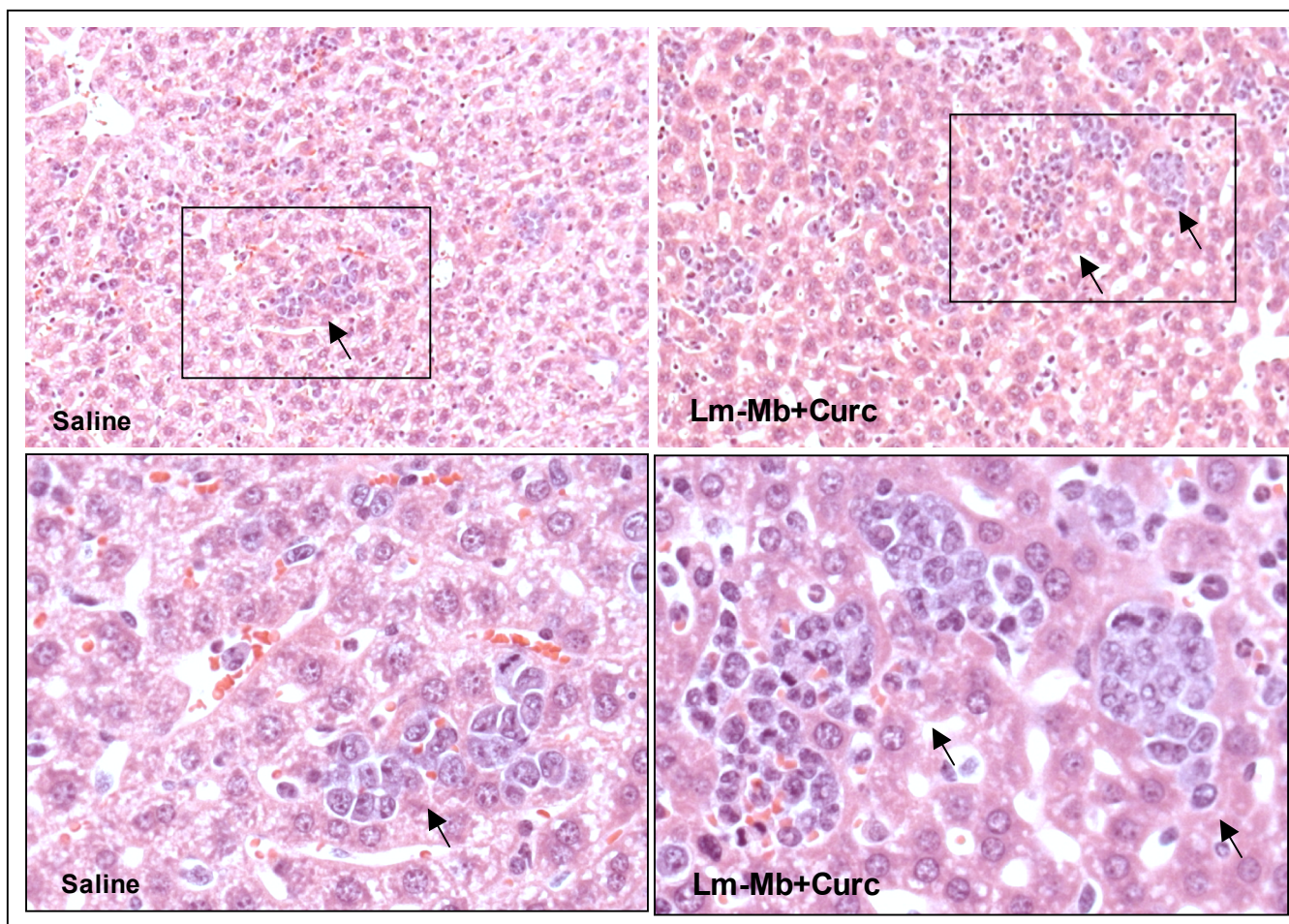

Supplement: Supplementary file 2 [file cam40002-0571-SD2.pdf]

Supplementary Information Figure S3

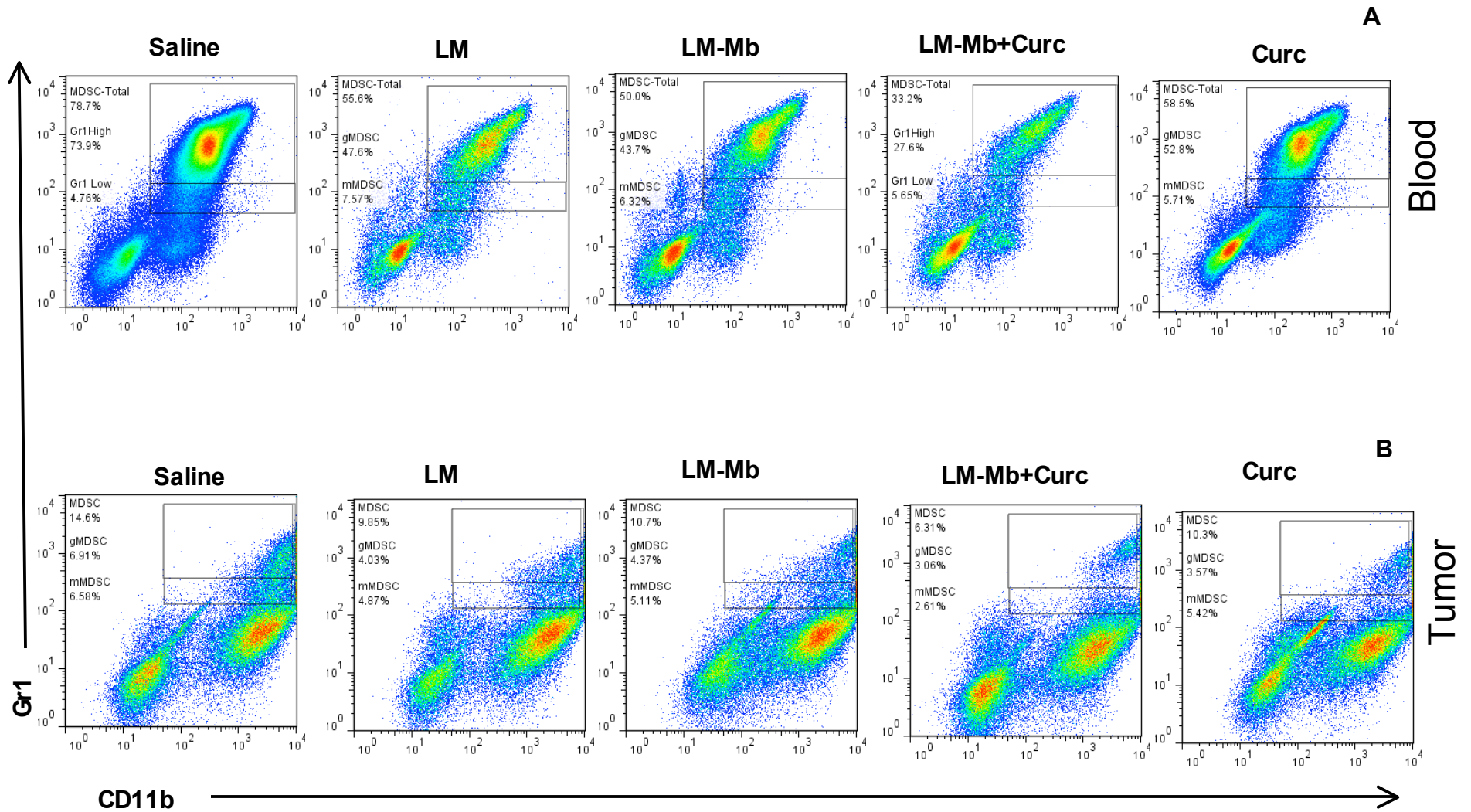

Supplement: Supplementary file 3 — Figure S3. The effect of Listeriaat-Mage-b and curcumin on MDSC in 4T1 tumor-bearing mice (Flow cytometry profile). [file cam40002-0571-SD3.pdf]
