# Supplementary material for: Curcumin improves the therapeutic efficacy of Listeriaat-Mage-b vaccine in correlation with improved T-cell responses in blood of a triple-negative breast cancer model 4T1
Source: Cancer Med. 2013 Jul 2;2(4):571–82. doi: 10.1002/cam4.94 (PMC3799292; doi:10.1002/cam4.94)
Supplement: Supplementary file 4 [file cam40002-0571-SD4.pdf]

Table S1: Histological examination of tissues after therapeutic treatment with Listeria^{at}-Mage-b and curcumin

	Mouse number					Mouse number				
**Organ**	**S1**	**S2**	**S3**	**S4**	**S5**	**MC1**	**MC2**	**MC3**	**MC4**	**MC5**
**Kidney**	nsf	nsf	nsf	nsf	nsf	nsf	nsf	nsf	nsf	nsf
**Heart**	nsf	nsf	nsf	nsf	nsf	nsf	nsf	nsf	nsf	nsf
**Lung**	icn3	icn1	icn2	icn3	icn1	icn3	icn3	icn1	icn3	icn3
**Liver**	cn0	cn0	cn0	cn0	cn0	cn1	cn0	cn0	cn0	cn0
**Liver**	emh3	emh0	emh2	emh3	emh1	emh4	emh4	emh1	emh2	emh3
**Liver**	ipp1	ipp0	ipp1	ipp1	ipp0	ipp2	ipp2	ipp0	ipp1	ipp2
**Liver**	gmi1	gmi0	gmi1	gmi1	gmi1	gmi2	gmi3	gmi0	gmi1	gmi0
**Spleen**	imh2	imh1	imh2	imh3	imh1	imh3-4	imh3-4	imh0	imh4	imh3-4

Nsf=no-significant finding, **icn**=increased circulating neutrophils, **cn**=coagulation necrosis, **emh**=extramedullary hematopoiesis, **ipp**=infiltrate, portal, mixed polymorphonuclear cells, **gmi**=granulomas, mixed inflammation, **imh**=increased myeloid extramedullary hematopoiesis
The numbers after the abbreviations represents the grade. 0=no finding, 1=minimal finding, 2= mild finding, 3=moderate finding, 4=marked finding, 5=severe finding. S=saline, MC=Listeria^{at}-Mage-b and curcumin.
