# Supplementary material for: Curcumin improves the therapeutic efficacy of Listeriaat-Mage-b vaccine in correlation with improved T-cell responses in blood of a triple-negative breast cancer model 4T1
Source: Cancer Med. 2013 Jul 2;2(4):571–82. doi: 10.1002/cam4.94 (PMC3799292; doi:10.1002/cam4.94)
Supplement: Supplementary file 5 [file cam40002-0571-SD5.doc]

**Figure S1: Significant reduction in the tumor size by preventive administration of curcumin followed by therapeutic immunization with Listeria^at^-Mage-b in 4T1 tumor-bearing mice.** BALB/c mice were treated with curcumin before tumor development and immunized with Listeria^at^-Mage-b after tumor development (Immunization protocol B), and analyzed for tumor weight during the treatments. This experiment was performed two times with 5 mice per group, and the results were averaged.

**Figure S2:** **The combination therapy of Listeria^at^-Mage-b and curcumin is non-pathogenic and non-toxic.** BALB/c mice were treated with curcumin before tumor development and immunized with Listeria^at^-Mage-b after tumor development (Immunization protocol B). Two days after the last immunization, mice were euthanized and liver sections were stained by H&E, followed by pathological examination. Increased myeloid extramedullary hematopoiesis (imh) (black arrow) was found in the liver of both groups, i.e. saline (negative control) and Listeria^at^-Mage-b and curcumin. The boxed areas in the top (magnification 200x) are shown in a larger magnification at the bottom (Light microscopy Magnification: 600x). Representative of two experiments.

**Figure S3: The effect of Listeria^at^-Mage-b and curcumin on MDSC in 4T1 tumor-bearing mice (Flow cytometry profile).** BALB/c mice were treated with curcumin before tumor development and immunized with Listeria^at^-Mage-b after tumor development (Immunization protocol B), and analyzed for MDSC (CD11b^+^Gr1^+^)**,** gMDSC (CD11b^+^Gr1^high^)**,** and mMDSC (CD11b^+^Gr1^low^) in blood **(A)** and primary tumors **(B)** using flow cytometry.
